# Musical Practice in Music Students During COVID-19 Lockdown

**DOI:** 10.3389/fpsyg.2021.643177

**Published:** 2021-05-17

**Authors:** Manfred Nusseck, Claudia Spahn

**Affiliations:** Freiburg Institute for Musicians' Medicine, Freiburg Centre for Research and Teaching in Music, Medical Faculty of the Albert-Ludwigs-University Freiburg, University of Music Freiburg, Freiburg, Germany

**Keywords:** formal practicing, self-efficacy, self-regulation, practice strategy, music students

## Abstract

The pandemic situation has forced students in higher education to use alternative learning routines due to reduced activities at universities and educational facilities. Especially music students needed to adapt their musical learning to this particular situation. Mostly affected by the lockdown was the musical practicing behavior, especially when practicing at the University of Music was not possible. In this study, music students in their second and third semesters were asked to provide information on their practicing situations during the coronavirus disease 2019 (COVID-19) lockdown. They were required to fill in questionnaires about the practicing time and concepts of self-efficacy and self-regulation for musical learning. The data of 18 music students were collected. For the analysis, they were compared with the answers of 15 music students who were asked the same questions half a year earlier before the pandemic situation occurred. The results showed that the music students relocated mostly to their parents' homes for practicing during the lockdown. In the amount of practicing, the bachelor of music students practiced less during lockdown compared with before the lockdown. The mean self-efficacy for musical learning did not differ between before and during the lockdown. For the self-regulated musical learning, the music students showed significantly higher values in the subscale on reflecting and creating a framework for the progress of musical learning during the lockdown. The findings indicate that the music students developed certain self-regulated learning skills during the lockdown and managed to find suitable solutions in continuing with their musical learning without reservation.

## Introduction

The coronavirus disease 2019 (COVID-19) pandemic resulted in severe restrictions during the summer semester 2020. In Germany, the government imposed certain requirements and regulations to prevent further spread of the coronavirus, the so-called lockdown. A necessary provision was to urge universities to cancel all live courses and events and to change to digital online teaching. For the students, this entailed no seminars and lectures with physical attendance, and they had to work at home. The teachers started to use formats of asynchronous learning, where the students had been given a task they could perform at their own pace until a certain deadline, or synchronous learning, where both students and teachers attended live online meetings. Thus, the students had to schedule their time more autonomously. An evaluation study with German students at universities showed that only a small amount of classes has been completely omitted and that the students managed the semester rather well with some constraints such as technical problems or individual stress experiences (Lörz et al., 2020). However, no evaluation has been made with music students addressing their specific study situation so far.

At the University of Music, the lockdown restrictions resulted additionally in the cancelation of orchestra, ensemble, and choir rehearsals and concerts. For music students, this is a rather large part of the curriculum. But most importantly, the individual instrumental and vocal lessons were affected, which are commonly taught in a one-on-one situation with teacher and student. For that, individual solutions for online teaching and asynchronous learning were found. Furthermore, the most essential learning activity of music students is practicing. During the lockdown, practicing was not allowed at the University of Music, and the music students needed to find other arrangements for musical learning than usually exist in a typical semester.

Music students devote tremendous amounts of time and energy to practice (Jørgensen and Hallam, 2016). It is driven by the understanding that practicing is the basis to attain technical and musical proficiency necessary to pursue a professional music career (Evans and Bonneville-Roussy, 2016). One of the main principles in achieving expertise is the concept of deliberate practice, which is described as practice activity following specific goals and self-monitored achievements (Ericsson et al., 1993; Lehmann and Ericsson, 1997).

The theoretical framework of Ericsson et al. (1993) postulates a close relationship between the amount of deliberate practice and individual level of expertise, indicating a high correlation between musical achievement and the time spent practicing in highly professional musicians. This finding, however, may suggest that musical achievement is more likely the result of one's efforts and quantity of time spent for practicing than of talents or gifts (Miksza, in press). Studies showed that practicing in long sessions can lead to exhaustion and limits in information processing and memory consolidation. An important emphasis should be given to rest and sleep (Ericsson et al., 1993; Cash, 2009). Additionally, a focus solely on practice time and excessive practicing may also result in severe health problems such as physical strain, overwork, and stress (Spahn, 2015).

Since higher levels of musical expertise were found to be associated with an increase in the adoption of systematic practice strategies (Hallam et al., 2018), recent research focuses on factors associated with the quality of practice to enhance the understanding of individual differences in performance levels. In a meta-analysis, Macnamara et al. (2014) found that despite the fact that deliberate practice is important for improving performance ability, it is not the only influencing factor. Thus, issues relating to the individual use of different practice strategies seem to play a crucial role (Jørgensen and Hallam, 2016).

There is a consensus in the literature concerning the conceptualization of practice time and that practicing should be divided into formal and informal practice time (McPherson and McCormick, 2006; Miksza, 2012). Bonneville-Roussy and Bouffard (2015) proposed a theoretical framework regarding formal practice and the associated factors predicting musical achievement. The core of the model constitutes the concept of formal practice, which mediates the influence of motivational factors and practice time on musical achievement. Formal practice is defined as goal-oriented and focused learning in combination with the use of deliberate practice and self-regulation strategies. In this model, practice time exerts a positive influence on the level of musical expertise only indirectly through the utilization of formal practice strategies. In accordance with the model, Hallam et al. (2012) found that the use of effective practice strategies and the amount of practice time increased with the level of expertise, while the use of ineffective strategies decreased. This implies that an individual set of practice strategies leads to reliable and consistent improvements in performance.

Musical learning involves a certain degree of autonomy (McPherson and Zimmerman, 2011). This includes having knowledge about the relevant parts that need to be learned and knowing how to organize and manage the practicing (Ritchie and Williamon, 2013). To achieve the practicing goal, musicians need to learn how to balance the effortful components of practice involving forethought, choice, and reflection.

Another factor influencing musical achievement is the concept of self-efficacy, which is strongly associated with formal and informal practice (McCormick and McPherson, 2003). Self-efficacy is conceptualized as the belief in one's own capacity to master a specific task (Bandura and Cervone, 1986). Moreover, an individual perception of competence influences the course of actions taken to accomplish a given problem. High self-efficacy therefore provides the possibility to use complex cognitive processes, to set individual goals, and to regulate stress in difficult situations for achieving successful results in particular tasks (Ritchie and Williamon, 2011). People with high self-efficacy tend to seek challenges, work harder, use different strategies, and persevere longer (Zimmerman, 2000). For learning, self-efficacy promotes the acquisition of relevant skills, knowledge, and structured learning approaches. In musical context, it leads to effective uses of formal practice strategies (Ritchie and Williamon, 2011). Furthermore, self-efficacy has been found to be a strong predictor for musical performance quality (McPherson and McCormick, 2006; Ritchie and Williamon, 2012).

A further important factor of formal practice is self-regulated learning (McPherson and McCormick, 1999). It describes an active participation in the learning process with initiating, choosing, and performing the practice in one's own way in contrast to just following external instructions (Ritchie and Williamon, 2013). Self-regulated learning includes having relevant schemata of what has to be learned and what has to be done to improve by identifying and correcting errors and by managing the practice and one's motivation (McPherson et al., 2018). It is related to perceived competence and musical performance achievements through formal practice (McPherson and McCormick, 2006). Self-regulation differs from deliberate practice, where learners use self-regulation strategies to increase experience. McPherson and McCormick (2006) argue that for practice time to be efficient, musicians need to have knowledge of what is necessary to improve and how they can achieve that goal. Otherwise, practice time might by reduced for building technical and motional automatisms.

Self-regulation has been shown to be correlated with the self-efficacy of learning (Ritchie and Williamon, 2013). Furthermore, advanced musicians have considerably higher self-regulatory skills, whereas less self-regulated learners rely more often on experienced others and social resources when practicing (McPherson and Zimmerman, 2011). Previous studies showed that music students practice on average about 3 h a day (e.g., Jørgensen, 2004). Nusseck and Spahn (2013) confirmed this practice duration in German music students and found that it was irrespective of the semester. However, they also showed that students pursuing bachelor's degree in music performance practiced significantly more than students who were studying to become school music teachers.

Besides practicing, music students also perform different kinds of physical activities, such as sports. A recent study on the effects of the lockdown on the well-being of performing artists found an increase in physical activities during the lockdown compared with before the lockdown (Spiro et al., 2021). In a previous study with German music students, Spahn et al. (2017) showed that in the first semester, 51% of the music students reported exercising at least once a week, and this amount increased up to 78% in the third semester 1 year later. It would be interesting to see if the increase in physical activities is even stronger during the lockdown.

Due to lockdown restrictions, music students had to adapt to new circumstances regarding their study processes and settings. A qualitative study investigated changes during the lockdown semester focusing on the relationship between music teachers and their students (Antonini Philippe et al., 2020). The authors found common themes of newly evaluating interpersonal relationships brought on by the distant contact. The music students were rethinking the relative importance of the relationship and were developing more self-involved attitudes with more autonomous behaviors, which resulted in a better understanding of their own learning. Despite these findings, little is known about differences in the practicing behavior during the lockdown compared with the usual semester.

The lockdown has challenged the music students by the absence of accustomed practicing routines and necessary facilities such as practicing rooms and may have caused particular adaptations in their practicing behavior. The lack of encounters with concerts and performances as well as instrumental and vocal lessons in online and asynchronous formats could have resulted in a decrease in or stagnation of confidence and motivation by the music students with possibly negative effects on formal practicing.

The special situation of the pandemic lockdown provided a unique opportunity to investigate direct impacts of an interruption in the University study on the practice behavior of music students. In our study, music students were asked about their practicing behavior during the lockdown, and their answers were compared with answers of students before the lockdown. In particular, the daily practice duration, the self-efficacy for learning, and aspects of self-regulated learning may differ between the lockdown and a typical semester. In addition, the students were also asked about where they practiced, when they usually practiced during the day, and how much other non-practice-related activities they performed. The main questions of this study were as follows:

- Did the mean practice time differ between before and during the lockdown?
- Are there differences in self-efficacy for musical learning between before and during the lockdown?
- Did the music students differ in self-regulated learning aspects before and during the lockdown?
- How did the practice situation, i.e., the location and the time of day, differ between before and during the lockdown?
- Are there changes in the amount of physical activities outside the field of music before and during the lockdown?

The overall aim of this exploratory study was to investigate aspects of practice behavior in music students focusing on differences between before and during the lockdown semester 2020. The lockdown situation has changed the daily routines drastically and affected the practice behavior of music students. The study goal was to examine how the students reacted in this situation. The results help to understand the circumstances under which music students practice and contribute to the discussion of the relevance of formal practice strategies.

## Materials and Methods

### Procedure

Two measurements were performed: the first in December 2019 (before the lockdown) and the second in June 2020 (during the lockdown). At the first measuring time, music students in their first semester of bachelor studies were asked for participation. At the second measuring time, music students in the second and third semesters have been considered, as first-semester students would have started their University study with the unusual lockdown semester.

At both times, the students were informed in an email about the study and were asked to fill in an online questionnaire. The web link has been provided in the email. The questionnaire was created with SoSci Survey. Considering the addressed population, about 50 music students were contacted at each measuring time.

The study has been approved by the Ethics Committee of the University of Freiburg.

### Participants

In total, 33 music students at the University of Music Freiburg participated in this study: 57.6% were female and 42.4% male (Table 1). The average age was 20.1 years (*SD* = 1.7 years). There were no significant differences in the mean age across gender. The music students were either studying bachelor of music with a focus on performance (45.5%) or bachelor with a pedagogical focus in teaching music in schools (school music, 54.5%). The musical instruments were dispersed in instrumental categories of 27% piano, 27% vocal, 27% wind, 13% strings, and 6% percussions. No significant distribution differences of the musical instrument categories were found for gender or study profile.

**Table 1:** Distribution and characteristics of the sample by measuring times (brackets, standard deviation).

	**Before the lockdown**	**During the lockdown**	**In total**
Amount (N)	15	18	33
Gender (in % female)	53.3%	61.1%	57.6%
Age (in years)	19.5 (1.3)	20.6 (1.8)	20.1 (1.7)
Studying bachelor of music	*N* = 8; 46.7%	*N* = 10; 44.4%	45.5%
Studying bachelor of music education	*N* = 7; 53.3%	*N* = 8; 55.6%	54.5%

At the first measuring time before the lockdown, 15 students participated in the survey. At the second measuring time regarding the time during the lockdown, 18 students took part in the study (Table 1). There were no significant distribution differences in the samples at both measuring times. Only the mean age was slightly higher in the second measuring time, *F*_(1, 31)_ = 3.425, *p* = 0.074, *d* = 0.67.

### Questionnaire

The first block of the questionnaire consisted of general questions such as gender, age, main instrument, and study profile. Additionally, the students were asked to estimate their mean practice time per day in minutes. After that, the standardized questionnaires described below have been included.

The second measuring time took place at the end of the hard lockdown when some restrictions had already been loosened. Therefore, in the introduction of the questionnaire, the music students were asked to relate their answers to the time of the strict lockdown. In the following, this measuring time will be described as during the lockdown.

#### Self-Efficacy for Musical Learning

The self-efficacy scale for musical learning from Ritchie and Williamon (2011) was used. For this study, the items were translated into German. The questionnaire consists of 11 items with a 7-point rating scale ranging from 1 (“not at all sure”) to 7 (“completely sure”). In a preliminary instruction, the respondents were asked to recall a usual performance or concert, imagine preparing for it, and respond to the statements with the current learning situation in mind. The higher the mean value of the scale, the higher the self-efficacy for musical learning. The self-efficacy scale showed with a single factor an internal consistency of Cronbach α = 0.82 (Ritchie and Williamon, 2011).

#### Self-Regulated Learning

For investigating aspects of self-regulated learning in musical context, the Musical Self-Regulated Learning Questionnaire of Ritchie and Williamon (2013) has been used. The questionnaire consists of 10 learning-related approaches to be rated as to how often the students do the described learning activity on a 7-point scale (1 = “not at all” to 7 = “always”). The items were translated into German for the purpose of this study. The questionnaire illustrates related aspects of different learning strategies on three different dimensions: (1) the scale Reflection considering the self-reflection of the practice process and the creation of frameworks for progress, (2) the scale Improvement focusing on the active pursuit of improvements inside and outside of practice, and (3) the scale Context addressing the setting of the learning context. The scale Reflection has been found to significantly correlate (*r* = 0.28) with the self-efficacy of musical learning (Ritchie and Williamon, 2013).

#### Questions Regarding the Practice Situation

The music students at the second measuring time during the lockdown were given additional questions regarding the practice situation. They were asked to report the frequency of practicing at specific locations and at certain times during the day on a 4-point scale (1 = “never” to 4 = “very often”). For the locations, the questions were about the University of Music, their own apartment, at the parents' home, and a specific rehearsal room. The given times during the day were in the morning, at midday, in the afternoon, and in the evening. The students were asked to provide answers referring to not only the current lockdown situation but also, in retrospect, the general situation before the pandemic.

#### Activities Outside of the Music Study

A particular block of questions considered the activities outside of the field of music, which has been taken from the Epidemiological Questionnaire for Musicians (EPI; Spahn et al., 2002). It addresses specific activities and the amount of regular exercises. The questions distinguish between four activity categories with general sports (such as jogging and endurance sport), relaxation techniques (such as autogenic training, yoga, and meditation), specialized body-oriented techniques (such as Alexander technique, Feldenkrais method, and dispokinesis), and psychologically oriented methods (such as mental training, supervision, self-reflection, and assertiveness training). The answers on the amount that each activity was performed were given on a 5-point scale, with 1 = “several times in a week,” 2 = “once a week,” 3 = “every 2 weeks,” 4 = “once a month,” and 5 = “never.”

### Data Analyses

SPSS (Version 26, Armonk, NY; IBM Corp.) was used for the data analysis. Descriptive statistics were calculated for each variable; and the mean with the standard deviation of the mean (*SD*) was reported. The Shapiro–Wilk test indicated that except for the practice time, the variables had a statistically normal distribution. For the parametric comparison, a one-way analysis of variance (ANOVA) was used to examine differences in all questionnaire scales and the practice time between the measuring times. The Levene *F* test revealed that the homogeneity of variance assumption was met. Differences between the study profiles at each measuring time were analyzed with independent *t*-tests. Contingency tables were used to assess the distribution differences of non-parametric variables, and chi-square statistics were stated. For the analysis of the changes in the practice location and time, Wilcoxon tests have been performed. Effect sizes were reported by using Cohen's *d* (Cohen, 1988). The level of statistical significance was set to 0.05.

## Results

Table 2 shows the mean values of all measures and the statistics of the ANOVA.

**Table 2:** Mean values of the questionnaire scales by measuring times (brackets, standard deviation; bold, *p* < 0.05; n.s., not significant).

	**Before the lockdown**	**During the lockdown**	**Statistics**
Practice time a day (in minutes)	136.0 (82.1)	128.6 (66.9)	*F*_(1, 31)_ < 1, n.s.
Self-Efficacy in musical learning	56.1 (7.5)	58.1 (10.3)	*F*_(1, 31)_ < 1, n.s.
Self-regulation scale Reflection	**3.4 (1.0)**	**4.3 (1.1)**	***F***_**(1, 31)**_ **=** **6.106**, ***p*** **= 0.019**, ***d*** **= 0.89**
Self-regulation scale Improvements	4.3 (1.2)	4.8 (0.9)	*F*_(1, 31)_ = 1.820, *p* = 0.187
Self-regulation scale Context	3.0 (1.1)	3.1 (1.1)	*F*_(1, 31)_ < 1, n.s.

**Figure 1:**
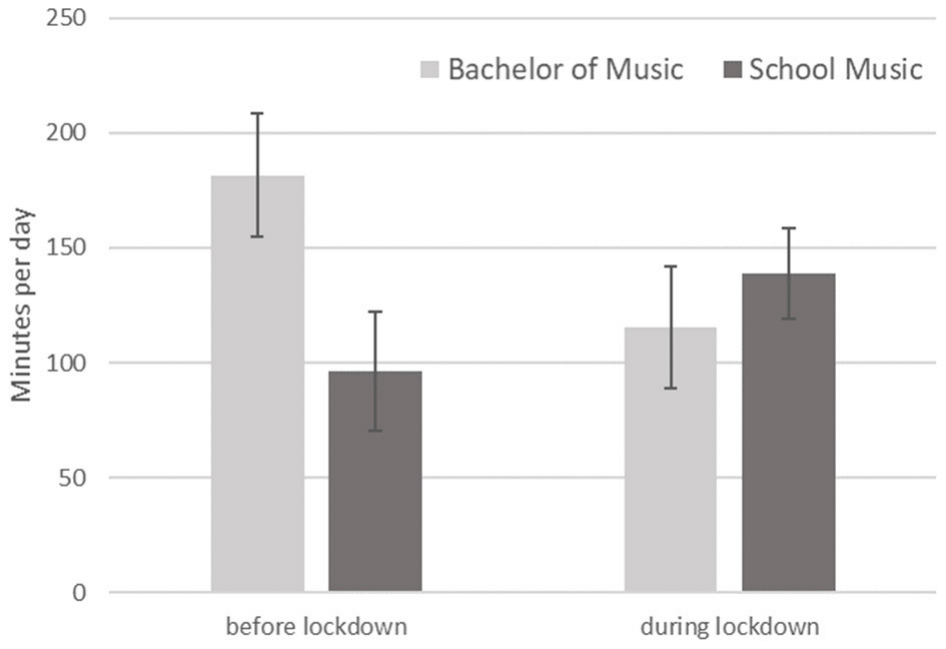
Mean practice times per day at the two measuring times split by study profile (error bars: standard error of the mean).

The mean practice time was not significantly different between the two measuring times. However, there was a significant difference of practice time between the studying profiles at the first measuring time, *t*(13) = 2.289, *p* = 0.039, *d* = 1.185, which was not found at the second measuring time, *t*(16) < 1.0. The mean practice time of both groups at the two measuring times is shown in Figure 1. Where the practice time per day of the music students with bachelor of music profile decreased from *M*1 = 181.4 min (*SD* = 70.8 min) to *M*2 = 115.6 min (*SD* = 74.9 min), the music education students increased practice time per day from *M*1 = 96.3 min (*SD* = 72.8 min) to *M*2 = 139.0 min (*SD* = 61.9 min).

The mean value of the self-efficacy scale in musical learning was not significantly different between the measuring times. Additionally, there was no significant difference found between the study profiles.

For the self-regulation scales, only the scale Reflection showed a significant difference between the measuring times with higher values during the lockdown. However, no significant difference was found for the study profile in any self-regulation scale.

There were also no significant correlations between the practice time and the self-efficacy and self-regulation scales.

**Figure 2:**
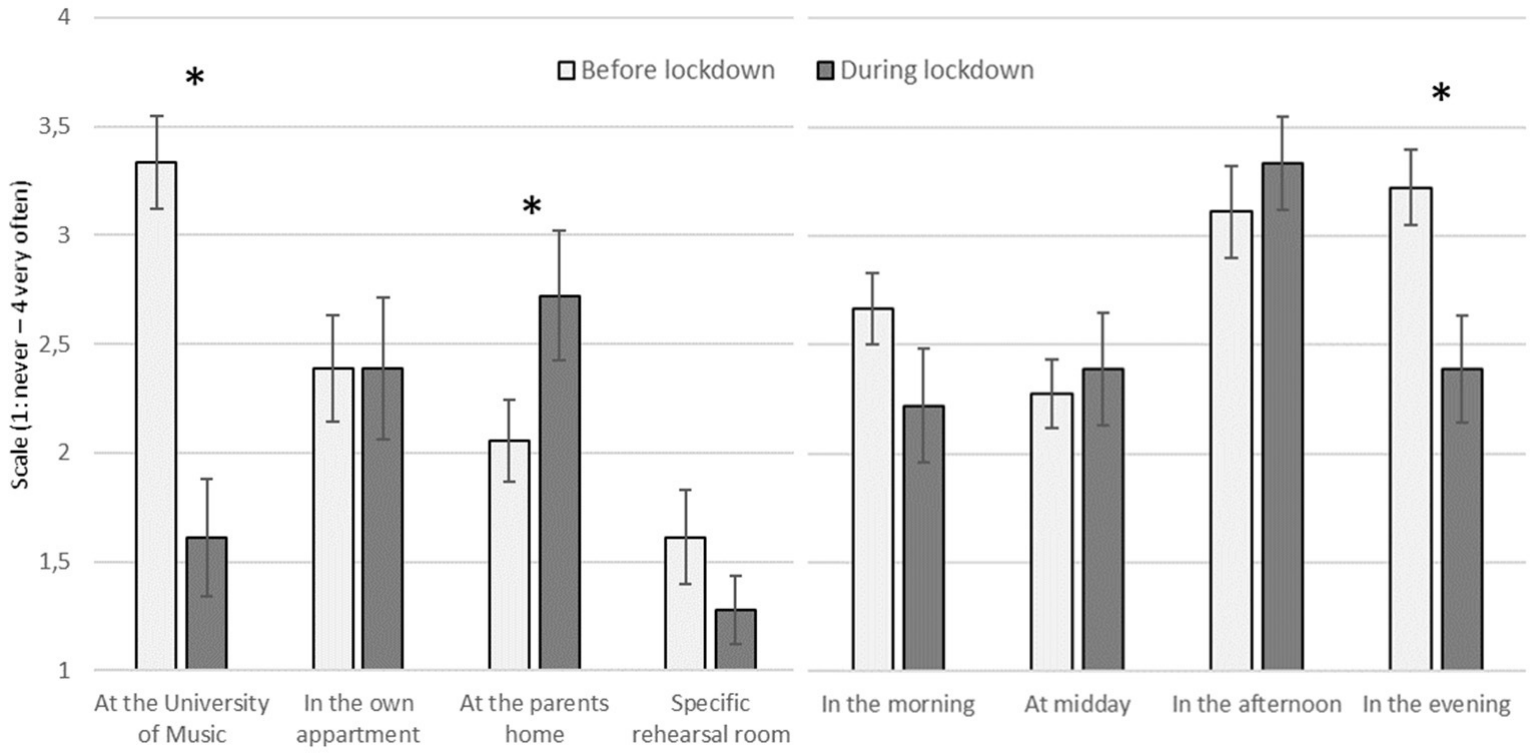
Mean values of the questions where and when the music students practiced before and during the lockdown (error bar: standard error of the mean; *p < 0.05).

In the answers to practice locations, there was a significant difference in the location at the University of Music, *Z* = −3.344, *p* = 0.001, *d* = 2.39, with less time practicing at the University of Music during the lockdown (Figure 2). For the location at the parents' home, a significant difference was found, *Z* = −2.041, *p* = 0.041, *d* = 1.06, with more time practicing at the parents' home during the lockdown.

The frequency of the practice time during the day showed only for the time in the evening a significant difference, *Z* = −2.565, *p* = 0.01, *d* = 1.46, with less evening practicing during the lockdown.

**Figure 3:**
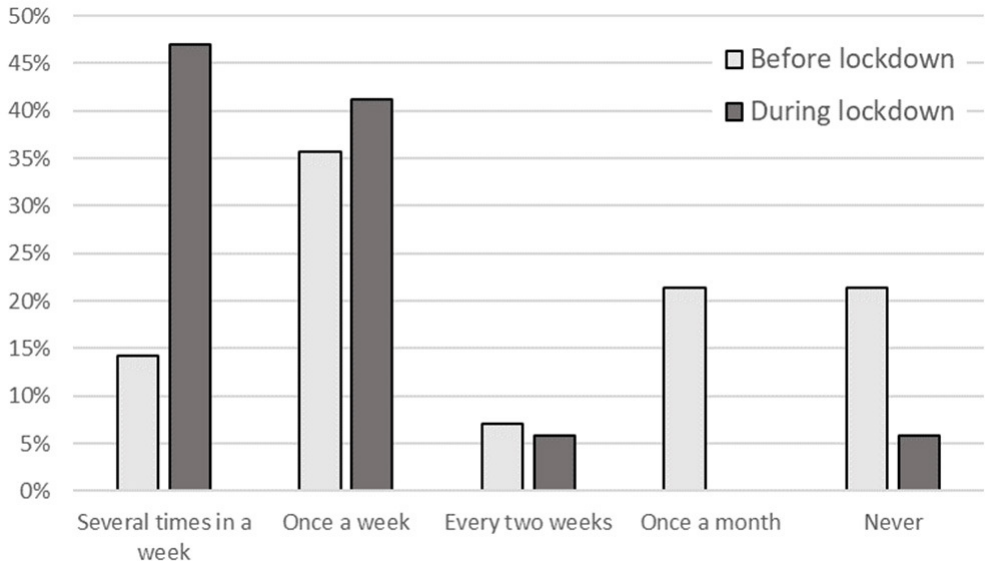
Histogram of the amount of general sports activities by measuring times.

For the physical exercises, a significant effect of time has been found for the general endurance sports activities, χ^2^ = 6.191, *p* = 0.013, *d* = 0.99. At the first measuring time, 50% of the music students performed endurance sports at least once a week (Figure 3). This amount increased up to 88.3% at the second measuring time during the lockdown. No significant differences have been found for studying profile and gender.

## Discussion

Under the very unique circumstances of the COVID-19 pandemic, music students were questioned about factors of formal practicing, such as the practice time, and about self-efficacy and self-regulated learning during the lockdown. The data were compared with those of music students who answered the same questions before the lockdown during a typical semester. Unfortunately, the sample size of the study was rather small. This limited the interpretations of the analysis results, and clear conclusions could only be made with caution. However, the results provided interesting aspects of how the students changed in certain practice behaviors, which can be related to the lockdown situation.

The average amount of time spent practicing per day was about 2 h. This was slightly lower than the average practice time found in previous studies (Jørgensen, 2004; Nusseck and Spahn, 2013; Spahn et al., 2017). The mean practice time did not differ between before and during the lockdown. This indicated that the music students practiced with the same amount before and during lockdown. However, there was a variance between the study profiles. The statistical results of the practice duration in both measuring times imply that there might be an interactive effect of the study profile. The bachelor of music (BA) students practiced before the lockdown about 2 h per day. This was rather similar to the findings for the BA students of the first semester in Spahn et al. (2017). Spahn et al. (2017) found that the practice time did not change in the following semesters: the BA students during the lockdown semester reported that their practice duration decreased at >1 h per day. This shows that the decrease in daily practice time could be an effect of the lockdown situation. It may be caused by the closing of the practice facilities or due to the missing pressure of routine upcoming concerts, auditions, and performances. This finding indicates that the BA students may have lost valuable practice experiences during the lockdown.

In contrast, the music students studying to become school music teachers practiced about 1.5 h per day before the lockdown and increased their practice time during the lockdown up to >2 h per day. Typically, music education students as opposed to the BA students have to attend several additional seminars and lectures regarding the curriculum for pedagogy and their second field of study. Therefore, it is known that the music education students have less time for practicing than the BA students. During the lockdown semester, in which these additional classes were either postponed or taught in online courses, less travel between different locations was required. The music education students seemed to use this time to practice more and even approached the level similar to that of the BA students.

If motivational aspects were responsible for the decrease in practice time of the BA students, a similar decrease in the self-efficacy scale for musical learning would be expected. However, there was no difference in the self-efficacy before and during lockdown, indicating that both BA and music education students maintained confidence about their practicing also through the lockdown semester. This discrepancy of reduced practice time and remaining perceived self-efficacy leads to the assumption that the students continued believing in their success in fulfilling their musical learning progress even with less amount of practicing.

Regarding the aspects of self-regulated learning, the study found that the music students increased in the scale Reflection during the lockdown independent from the studying profile. This scale focuses on the own beliefs in decision making and choosing courses of action and specifically in self-evaluating and on the individual setting of goals for the practice target (Ritchie and Williamon, 2013). It was shown that the music students increased practicing in a more self-monitored way. This is understandable considering the reduced amount of instrumental and vocal lessons. The students had to supervise their own practicing process and outcome. This is an important factor in self-regulated learning and supports a successful learning strategy. The finding therefore indicates that the students seem to rely usually more on the teachers' advice than on their own beliefs. Due to the lockdown, the students seem to have developed this self-regulatory skill that is comparable with that of advanced musicians (McPherson and Zimmerman, 2011). Following this kind of increased autonomy, it leads to a better understanding of the individual learning process and provides lasting forms of learning (McPherson and McCormick, 1999).

The findings of the increased self-regulated skill also confirm the findings of Antonini Philippe et al. (2020) that the music students reported of rethinking the relationship with their music teacher and used more self-involved attitudes and autonomous behaviors. Thus, the lockdown seems to have provided a platform for developing a better understanding of one's own learning.

Another particular aspect of interest was the practice location in the lockdown semester. It was to be expected that the practice time at the University of Music was drastically reduced during the lockdown. However, the music students seem to have found reliable solutions, and most of them used their parents' home for practicing. As some instruments are difficult to practice in their own apartment, the unchanged amount of practicing there indicates that the same students were still practicing in their own apartment as before the lockdown.

The usual time for practicing during the day did not significantly change during lockdown, despite the apparent decrease in practicing in the evening. This could be caused by the fact that there were no lessons or rehearsals during the day, which may usually force the students to practice more in the evening. However, since the other times during the day were similarly occupied before and during the lockdown, the students seemed to focus their practicing mostly on these times, resulting in a free evening. The students practiced more during the day and used the evening for a break in practicing. From a health perspective for musicians (Spahn, 2015) recommending a focus on rest and sleep, this indicates a good way of practicing.

The question as to what the music students actually did in the evening cannot be answered, but the findings in the answers to the physical activities may provide a hint. The execution of endurance sports increased drastically. This confirms the findings of Spiro et al. (2021). Before the lockdown, 50% of the students answered that they do sports at least once a week, which was very similar to the findings of Spahn et al. (2017). The amount of students performing sports increased up to 88% during the lockdown. This is even more than reported in Spahn et al. (2017), indicating that more students were doing sports during the lockdown semester than usual. This implies that the music students used the time not spent on traveling to University locations to also improve physical stamina and followed the recommendations of musician's health (Spahn, 2015) for doing various preventive activities regarding physical exercises. As Spiro et al. (2021) also found relations between positive well-being and physical activities, this may therefore be a positive health effect.

### Limitations of the Study

The present study had some limitations regarding the methodology. A particular limitation of this study was the sample size. Despite the clearly existing statistical results, a larger sample would have provided a more detailed analysis and the potential to differentiate across the instruments. A possible increase in the sample sizes could only have been implemented by performing the survey as a multicenter study at different Universities of Music. Nevertheless, as the COVID-19 lockdown was (hopefully) a one-time situation, the study is not reproducible but provides relevant insights into the practice behavior of music students.

## Conclusion

The findings provide certain understandings for the circumstances of practicing in music students. The lockdown situation forced both students and teachers to adapt their learning and teaching approaches. The typical one-on-one situation in instrumental and vocal lessons needed to be shifted to online classes and asynchronous learning formats. These changes created some spatial and time freedom for the students and led to an increase of their autonomy. The music students seem to have developed an individual and more self-motivated level of practicing and used their efforts more effectively to accomplish their practicing goals.

This specific transition during the lockdown semester could be used to rethink the way of teaching instrumental and vocal lessons. It showed that the change from a more teacher-mediated method of instructing and advising to a more learner-oriented method increased important skills of self-regulated learning and shifted the source of knowledge back to the student.

## Data Availability Statement

The raw data supporting the conclusions of this article will be made available by the authors, without undue reservation.

## Ethics Statement

The studies involving human participants were reviewed and approved by Ethical Commission of the University of Freiburg. Written informed consent for participation was not required for this study in accordance with the national legislation and the institutional requirements.

## Author Contributions

All authors contributed extensively to the work presented in this paper. They contributed to the conception, design of the study, data collection, and statistical analyses. The authors co-wrote the manuscript.

## Conflict of Interest

The authors declare that the research was conducted in the absence of any commercial or financial relationships that could be construed as a potential conflict of interest.
